# Commentary: Observational Behavior Assessment for Psychological Competencies in Police Officers: A Proposed Methodology for Instrument Development

**DOI:** 10.3389/fpsyg.2021.686576

**Published:** 2021-07-12

**Authors:** Mario S. Staller, Swen Koerner

**Affiliations:** ^1^Department of Policing, University of Applied Sciences for Police and Public Administration in North Rhine-Westphalia, Aachen, Germany; ^2^Department of Training Pedagogy and Martial Research, German Sport University Cologne, Cologne, Germany

**Keywords:** assessment instrument, behavioral observation, police, police training, expertise-oriented approach, professional performance

Koedijk et al. recently proposed a step-by-step methodology for police organizations to develop an observational behavior assessment instrument to assess psychological competencies of police officers in a meaningful way. We strongly appreciate widening the scope of the assessment of psychological competencies beyond the pure measurements of constructs *via* self-reports and questionnaires toward measuring performance within representative scenarios. However, we think the proposed approach is still reductionistic in its scope resulting in various problems as far as it concerns the development of adaptive and flexible police officers.

Koedijk et al. build their assessment approach on the assumption that police officers need to develop a certain level of competence, that can then be evaluated through observable competencies. While broadly competence refers to the practitioner's integrated skills (Fouad et al., 2009), the construct of expertise goes beyond that (Cruickshank et al., 2018; Staller et al., 2021a). In contrast to competencies that can be observed in techniques and behaviors, expertise centers on the ability to make sound judgements to identify and deploy the optimum blend of techniques and behaviors to meet the demands of complex and dynamic situations in the field (Cruickshank et al., 2018). Especially, in the dynamic context of interpersonal interaction like in the domain of policing a more nuanced approach of evaluation that goes beyond right or wrong is needed. Such an approach needs to focus on cognition (i.e., perception, attention, reasoning, problem solving, and decision-making), and an “it depends”-approach to assess the appropriateness of performance. As such questions like “What options did you consider and why did you choose a specific one over others?” shed light on the cognitive processes underlying the observed behavior, thus going beyond the dichotomy of “desirable” or “problematic” behavior.

In the light of nuanced solutions to complex problems, the development of reflexivity seems to be a key goal for police officers that may best be achieved by the adoption of an expertise-oriented approach to training and evaluation (Staller et al., 2021a,b). Reflexivity refers to reflecting on performance on different levels and as such uncovering performance-underlying assumptions about what is perceived as right or wrong. Also, concerning the expectations of specific behaviors, a pre-determined approach to what constitutes right or wrong behaviors does not account for the ecological dynamics of functional behaviors. Within the framework of ecological dynamics problem solving is viewed as the realization of perceived affordances within the performer-environment system (Araújo et al., 2017); emergent behavior resulting from the continuous interaction between individual, task, and environmental constraints will likely result in new solutions to problems that have not been accounted for before (Koerner et al., 2021).

Reflexivity and ecological cognition cannot be measured solely by the outputs (i.e., the observed behaviors). Instead, emphasis has to be put on the thinking and decision-making process underlying these behaviors. In this way, a focus on the thinking process aids the development of declarative over procedural knowledge (Cruickshank et al., 2018). These declarative knowledge structures drive the ability to adapt to new contexts, cope with complex and unfamiliar tasks, generate new knowledge, and continue to raise the individual level of performance (Anderson, 1982).

Police organizations neglecting the underlying thinking processes when assessing observed behavior may nurture problematic aspects concerning the training and education of police officers (Koerner and Staller, 2020; Koerner et al., 2021). These include

- police officers training for the test instead for performance in the field by focusing on the desired output instead of the underlying production process of any behavioral solution,
- police trainers focusing on the to be shown competencies, thus neglecting a focus on problem-solving processes of their learners, and
- a neglection in the development and assessment of individual solutions to problems, that emerge as a result of the individual problem-solving process.

Taken together, we would like to encourage the authors—and police organizations—to refrain from a competence-oriented approach, that underlies the conceptualization of the proposed assessment (Fouad et al., 2009), and adopt an expertise-oriented framework (Collins et al., 2014) embedded within an ecological approach on cognition and functional decision making (Araújo et al., 2017).

## Author Contributions

MS and SK authors contributed equally to the ideas presented. MS wrote the first draft of the paper. Both authors contributed equally to editing the first draft to its final version.

## Conflict of Interest

The authors declare that the research was conducted in the absence of any commercial or financial relationships that could be construed as a potential conflict of interest.
